# APPLYING THE CARE SETTING TRAJECTORY DATABASE TO STUDY END-OF-LIFE OUTCOMES IN PERSONS WITH DEMENTIA

**DOI:** 10.1093/geroni/igad104.1467

**Published:** 2023-12-21

**Authors:** Hyosin Kim, Olga Jarrín, Paul Duberstein, Bei Wu, Haiqun Lin, Anum Zafar, S Marie Harvey, Maria Lopez

**Affiliations:** Oregon State University, Corvallis, Oregon, United States; Rutgers, The State University of New Jersey, New Brunswick, New Jersey, United States; Rutgers School of Public Health, Piscataway, New Jersey, United States; New York University, New York City, New York, United States; Rutgers, The State University of New Jersey, Newark, New Jersey, United States; Rutgers, The State University of New Jersey, New Brunswick, New Jersey, United States; Oregon State University, Corvallis, Oregon, United States; Rutgers, The State University of New Jersey, New Brunswick, New Jersey, United States

## Abstract

This study highlights an application of the care setting trajectory database and our new, harmonized, self-reported race/ethnicity variable to evaluate the impact of home health care on later use of hospice and place of death in a large cohort of Medicare decedents. This study evaluated home health care use during the last 3 years of life as a predictor of death in the care of hospice for a cohort of 895,526 Medicare beneficiaries with dementia who died in 2019. The cohort was limited to beneficiaries continuously enrolled for at least 5 years, who were age 40 and older, and who lived in the U.S. fifty states, District of Columbia, or Puerto Rico at death. Co-variates included number of acute hospitalizations during the last 3 years of life, age at death, sex, race/ethnicity, comorbidities, insurance, rural/urban area, and state of residence. 54.5% of the cohort used home health care at any point during the last 3 years of life. Compared to beneficiaries who did not use home health care, those who received home health care were more likely to die at home (64.5% vs. 60%, OR 1.08) or in the care of hospice (65% vs. 58%, OR 1.05). Among beneficiaries who died with hospice, use of home hospice was more prevalent for those who had previously used home health care (53% vs. 49%). This study provides compelling evidence of the value of home health care as a strategy to improve end-of-life care quality among Medicare beneficiaries living with dementia.

